# Activity of NAD(P)H-Oxidoreductases in Ovarian Cancer

**DOI:** 10.3390/biomedicines12051052

**Published:** 2024-05-10

**Authors:** Maria V. Fedorova, Vladimir I. Voznesensky, Elena A. Sosnova, Elena V. Proskurnina

**Affiliations:** 1 Central Research Institute of Epidemiology, Federal Service for Surveillance on Consumer Rights Protection and Human Wellbeing, 111123 Moscow, Russia; theklazontag@yandex.ru; 2 Pletnev Moscow City Clinical Hospital, Moscow Department of Health, 105077 Moscow, Russia; vlad525@gmail.com; 3 Department of Obstetrics and Gynecology No. 1, Sechenov First Moscow State Medical University of the Ministry of Health of the Russian Federation (Sechenov University), 119048 Moscow, Russia; sosnova_e_a@staff.sechenov.ru; 4 Research Centre for Medical Genetics, 115522 Moscow, Russia

**Keywords:** cytochrome b5 reductase, cytochrome P450 reductase, ovarian cancer, peritoneal fluid, antioxidant capacity

## Abstract

Reactive oxygen species (ROS) play an important and controversial role in carcinogenesis. Microsomal redox chains containing NADH- and NADPH-dependent oxidoreductases are among the main sites of intracellular ROS synthesis, but their role in the oxidative balance has not been fully studied. Here, we studied the activity of cytochrome b5 reductase (CYB5R) and cytochrome P450 reductase (CYPOR) in ovarian cancer tissues and cells isolated from peritoneal fluid, along with the antioxidant capacity of peritoneal fluid. We used the developed a chemiluminescence assay based on stimulation with NADH and NADPH, which reflects the activity of CYB5R and CYPOR, respectively. The activity of CYB5R and CYPOR was significantly higher in moderately and poorly differentiated ovarian adenocarcinomas compared with well-differentiated adenocarcinomas and cystadenomas. For the chemotherapy-resistant tumors, the activity of tissue CYB5R and CYPOR was lower compared to the non-resistant tumors. In the peritoneal fluid, the antioxidant capacity significantly increased in this series, benign tumors < well-differentiated < moderately and poorly differentiated adenocarcinomas, so the antioxidant excess was observed for moderately and poorly differentiated adenocarcinomas. The antioxidant capacity of peritoneal fluid and the activity of CYB5R and CYPOR of cells isolated from peritoneal fluid were characterized by a direct moderate correlation for moderately and poorly differentiated adenocarcinomas. These results indicate the significant role of NAD(P)H oxidoreductases and the antioxidant potential of peritoneal fluid in cancer biochemistry. The parameters studied are useful for diagnostics and prognostics. The developed assay can be used to analyze CYB5R and CYPOR activity in other tissues and cells.

## 1. Introduction

Ovarian cancer is the eighth most common cancer in women, accounting for an estimated 3.7% of cases and 4.7% of cancer deaths in 2020. Ovarian cancer is the third most common gynecological cancer and has an incidence of approximately 308,069 cases worldwide (2020) with very high mortality rates [1]. The death rate for ovarian cancer is three times higher than for breast cancer and is projected to increase significantly by 2040 [2]. Ovarian cancer is a very heterogeneous disease, and, even among epithelial ovarian cancer, there are five major histotypes. Serous adenocarcinoma is the most common subtype of ovarian cancer [3]. The lack of specific symptoms, especially in the early stages of the disease, together with the heterogeneity and resistance to therapy, reduces the life expectancy of patients. Despite a huge number of studies, there is an actual necessity to discover more diagnostic markers [4].

The intracellular mechanisms of ovarian carcinogenesis are multifactorial, so the treatment strategy includes chemotherapy, immunotherapy, and therapy aimed at neo-angiogenesis [5,6]. Drug resistance is a bottleneck in ovarian cancer treatment despite the application of new drugs. A profound understanding of the mechanisms of resistance is necessary to solve this problem [7]. The main mechanisms of resistance are disorders in transmembrane transport and in DNA damage repair, the dysregulation of cancer-associated signaling pathways, and epigenetic modifications such as DNA methylation, histone modifications, and non-coding RNA activity. One drug may have several mechanisms of resistance. Conventional chemotherapy drugs and targeted drugs may have cross-resistance mechanisms. Specialists highlight the urgent need to develop biomarkers for the chemoresistance and prognosis in ovarian cancer [8,9].

Carcinogenesis is a complex, multicomponent, and not fully understood process, in which oxidative metabolism plays a paradoxical role [10]. On the one hand, reactive oxygen species (ROS) are needed for cancer cell proliferation, metastasis, and chemoresistance. On the other hand, high levels of ROS have cytotoxic and pro-apoptotic effects [11]. ROS control the signaling pathways, transcription factors, genes, and tumor microenvironment [12]. In patients with chemoresistance, cancer cells are protected from death with the marked expression of ROS-dependent genes [13]. Interestingly, excess antioxidants can also be a basis of radio- and chemoresistance [14].

Microsomal redox chains are significant sites of intracellular ROS metabolism. Microsomal reductases built into the endoplasmic reticulum membrane transport electrons from NADH or NADPH to the cytochromes b5 and P450. In addition to the main function, NADH-dependent cytochrome b5 reductase (CYB5R) takes part in the synthesis of cholesterol, elongation of fatty acids, and microsomal hydroxylation of xenobiotics and steroid hormones. It is a part of the transmembrane redox system, which maintains ascorbate and coenzyme Q10 in a reduced state, thus protecting cells from apoptosis [15]. The NADH-dependent redox chain is located also in the outer mitochondrial membrane. The role of CYB5R in carcinogenesis is not well-understood. Its increased expression correlates with a poor prognosis in patients with estrogen-receptor-negative breast cancer. A decreased CYB5R expression significantly reduces metastasis to the lungs in a mouse model [16].

The NADPH-dependent cytochrome P450 reductase (CYPOR) transfers an electron to cytochrome P450, cytochrome b5, heme oxygenase, squalene monooxygenase, and 7-dehydrocholesterol reductase. The most important function of the NADPH-dependent cytochrome P450 chain is the metabolism of prodrugs [17,18]. Regarding the role of CYPOR in cancer biochemistry, most studies are devoted to the metabolism of anticancer drugs [19,20]. The deficiency of CYPOR function contributes to the resistance to antifungal drugs [21,22] and doxorubicin [23]. Interestingly, the suppression of CYPOR results in the resistance to ferroptosis, which is an important mechanism of cell death in cancer [24]. We hypothesized that the activity of this enzyme may be important in chemoresistance in ovarian cancer.

Peritoneal fluid is involved in carcinogenesis in ovarian cancer. Cancer cells, entering the peritoneal fluid, create special conditions for their survival, proliferation, and metastasis [25,26,27]. Peritoneal fluid in ovarian cancer contains increased concentrations of HGF (hepatocyte growth factor), TGF-β1 (transforming growth factor beta-1), and GRO-1 (growth-related oncogene-1). HGF and GRO-1 promote the cellular senescence of mesothelial cells and increase the production of hyaluronic acid, u-PA (urokinase plasminogen activator), IL-8 (interleukin-8), and MCP-1 (monocyte chemotactic protein-1), which stimulate cell adhesion, proliferation, and migration in ovarian cancer [25]. The senescence of mesothelial cells in ovarian cancer is associated with oxidative damage to DNA and lipids due to oxidative stress. At the same time, the activity of cytochrome c oxidase and NADH dehydrogenase decreases, and the potential of the inner mitochondrial membrane decreases [28]. Highly aggressive and undifferentiated tumor cells in the peritoneal fluid are more capable of metastasis, which is associated with the increased production of ROS mediated by p38 mitogen-activated protein kinase and nuclear factor NF-κB [26]. Biochemical parameters of peritoneal fluid, such as IL-6, may be a valuable diagnostic marker for ovarian cancer [29].

Thus, we aimed to study the activity of CYB5R and CYPOR in ovarian cancer tissues from patients, as well as to evaluate the prospects for the chemiluminescent analysis of cells from peritoneal fluid. A promising method for studying the activity of CYB5R and CYPOR is lucigenin-enhanced chemiluminescence stimulated by NADH and NADPH, respectively. Lucigenin is directly reduced by CYB5R and CYPOR. In the presence of oxygen, a superoxide anion radical is formed. As a result, chemiluminescence occurs, which is proportional to the activity of CYB5R and CYPOR [30,31,32,33]. We have developed an assay and successfully used it to study the activity of NAD(P)H oxidoreductases in thyroid tumors [34]. We have shown that the activity of CYB5R and CYPOR may serve as promising biomarkers for differential diagnostics of benign and malignant thyroid tumors. We also examined the activity of CYPOR and CYB5R in cervical and endometrial cancer and showed that the activity differs for moderately differentiated and poorly differentiated adenocarcinomas [35]. These data indicate the significant role of NAD(P)H oxidoreductases in cancer biochemistry.

Here, we studied the activity of CYB5R and CYPOR in ovarian serous adenocarcinoma as the most common subtype of ovarian cancer using our original assay and compared these data with the resistance to chemotherapy. We analyzed both tissue samples and cells isolated from peritoneal fluid. We also studied the antioxidant capacity of peritoneal fluid with an original chemiluminescence assay for a more comprehensive understanding of the oxidative balance in the abdominal cavity.

## 2. Materials and Methods

### 2.1. Patients

An observational, single-stage, uncontrolled, single-center pilot study included 42 patients with ovarian cancer (age from 47 to 72 years). The cases involved well-, moderately, and poorly differentiated serous adenocarcinoma (T1aN0M0 to pT3cNXM1). The control group was represented by 11 patients with benign ovarian tumors (serous cystadenoma). Exclusion criteria were as follows: age over 75 years and other malignant tumors. All patients signed informed consent for inclusion in the study. The study was conducted in accordance with the Declaration of Helsinki, and approved by the Ethics Committee of the Research Centre for Medical Genetics (Approval #5, 3 July 2017).

### 2.2. Preparation of Tissue and Peritoneal Fluid Samples

Surgery and observation of patients were carried out at the Pletnev City Clinical Hospital (Moscow, Russia). Histological verification was performed at the Pathology Department of this hospital. Tissue samples were transported in 0.9% NaCl at +4 °C. Peritoneal fluid samples were transported in a vacutainer with Li-heparin at +4 °C and analyzed no later than two hours after taking the samples. The volume of peritoneal fluid from each patient was at least 20 mL. All samples were analyzed no later than two hours after taking.

### 2.3. Assessment of Tissue NAD(P)H Oxidoreductases with Chemiluminescence

To assess the activity of CYB5R and CYPOR, a protocol was developed based on lucigenin-enhanced chemiluminescence of tissue stimulated by NADH or NADPH, respectively. Chemiluminograms were recorded on a 12-cuvette Lum-1200 chemiluminometer with Power Graph 3.0 software (DISoft, Moscow, Russia). NADH- and NADPH-dependent chemiluminescence in triplicates was recorded simultaneously for each sample, which ensured high reproducibility and comparability of chemiluminograms.

A buffer Krebs–Ringer solution (pH 7.4) was prepared on the day of the experiment. Solutions of lucigenin (1mM, 10,10-dimethyl-9,9-biacridinium dinitrate), NADH, and NADPH (10 mM) were prepared by dissolving weighed samples in deionized water. All reagents were purchased from Sigma-Aldrich, St. Louis, MO, USA.

Tissue samples were washed three times with a Krebs–Ringer solution. Six portions (15.5 ± 0.5 mg) were taken from each sample with a 20G biopsy needle (GTA, Quistello, Italy). The samples were placed in cuvettes containing a Krebs–Ringer solution (1860 μL) and 1 mM lucigenin solution (120 μL). Chemiluminescence was recorded at 37 °C for 10 min; then, 10 μL of 10 mM NADH or NADPH was added. The signals were recorded for another 20 min. From the chemiluminograms, we calculated the intensity of the basal luminescence *I*_0_, the intensity of the stimulated luminescence *I*_NADH_ and *I*_NADPH_, and the activation coefficients *K*_NADH_ = (*I*_NADH_ − *I*_0_)/*I*_0_ and *K*_NADPH_ = (*I*_NADPH_ − *I*_0_)/*I*_0_.

### 2.4. Assessment of NAD(P)H Oxidoreductases in Cells Isolated form Peritoneal Fluid

We applied our assay to analyze CYB5R and CYPOR activity in ascitic fluid cells. A 10 mL aliquot of peritoneal fluid was centrifuged for 40 min at 2000× *g*. The cell pellet was washed with 10 mL of a Krebs–Ringer solution, centrifuged for 20 min, and the procedure was repeated twice. Next, 1 mL of Krebs–Ringer solution was added to the cell pellet. From this sample, 9 portions of 100 μL were added to cuvettes containing 120 μL of 1 mM lucigenin. In the last portion, cells were counted using a flow cytometer (CytoFlex S, Beckman Coulter, Brea, CA, USA). Chemiluminescence was recorded at 37 °C for 5 min; then, 10 μL of 10 mM NADH or NADPH was added in triplicates and the signals were recorded for another 10 min. Three samples without the addition of NADH and NADPH served as blank. The chemiluminescence of the supernatant peritoneal fluid was measured in a similar manner. From the chemiluminograms, we determined the intensity of the basal luminescence *I*_0_, the intensity of the stimulated luminescence *I*_NADH_ and *I*_NADPH_, and the activation coefficients *K*_NADH_ = (*I*_NADH_ − *I*_0_)/*I*_0_ and *K*_NADPH_ = (*I*_NADPH_ − *I*_0_)/*I*_0_. *I*_NADH_ and *I*_NADPH_ were normalized to the number of cells in the cuvette.

### 2.5. Chemiluminescence Assay for Antioxidant Capacity of Peritoneal Fluid

**Figure 1:**
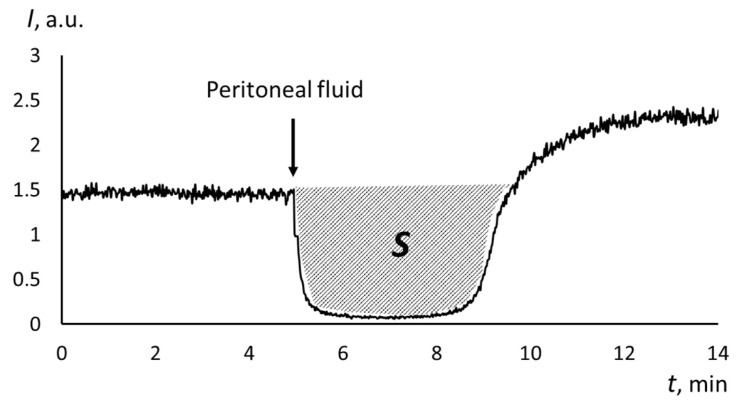
The chemiluminogram for the peritoneal fluid of the patient with cystadenoma; *S* is the luminescence suppression area, which is proportional to the water-soluble antioxidant capacity. The arrow indicates the moment of adding the peritoneal fluid.

Chemiluminescence was recorded at 37 °C in a system containing a free radical generator 2,2′-azo-bis(2-amidinopropane) dihydrochloride (ABAP) and luminol in a 100 mM phosphate buffer solution (PBS, pH 7.4) (all reagents from Sigma-Aldrich, St. Louis, MO, USA). The luminescence was recorded until the plateau was reached, then a 10 μL aliquot of peritoneal fluid diluted 10 times with PBS was added. The luminescence was recorded until a new plateau was reached. With the PowerGraph 3.0 software (DISoft, Moscow, Russia), the luminescence suppression area *S* was calculated, which is proportional to the antioxidant capacity of water-soluble antioxidants (Figure 1). The reference limits for blood plasma were determined previously (*n* = 98) as 195–405 a.u. [36]. A decrease in *S* corresponds to oxidative stress.

### 2.6. Chemiluminescence Assay for Studying Effects of Uricase in Antioxidant Capacity

To clarify the contribution of uric acid to the antioxidant capacity, we used a test with uricase. A 5 mg portion of uricase (Uricase from *Bacilius fastidiosus*, #94310, 13.4 U/mg, Sigma, St. Louis, MO, USA) was dissolved in 500 μL of phosphate buffer solution (100 mM, pH 7.4). A uricase working solution was prepared by diluting the stock solution 100 times with PBS. By definition, 1 U of uricase activity oxidizes 1 mmol of uric acid at pH 9.1 and 37 °C. Although the optimal is pH 9.2, uricase activity remains high at pH 7.4 [37]. The upper reference limit for uric acid in blood is 420 µmol/L. Taking into account a hematocrit of 50%, the concentration can be taken for ease of calculation as 1000 µmol/L (1 mmol/L). The cuvette contains 1 μL of plasma that corresponds to 1 nmol of uric acid, which requires 1 nanounits of uricase for oxidation. Experimentally, we found the conditions for complete suppression of antioxidant capacity in blood plasma. Taking into account possible enzyme inhibition and other factors, we used an excess of enzyme activity of 1.3 × 10^7^ times. To sum, a 10 μL aliquot of uricase working solution (a final concentration of 0.0134 U/mL that corresponds to 0.0134 U in the cuvette) was added to the cuvette containing 10 μL of peritoneal fluid diluted 10 times with PBS, shaken, incubated for 15 min in the dark at 37 °C, and analyzed as described above.

### 2.7. Statistics

All measurements were performed in triplicate. The data are presented as mean and standard deviation. The normality of distribution was checked using the Shapiro–Wilk test. A comparative analysis of two independent groups was carried out using the Mann–Whitney test. Differences were considered statistically significant at *p* ≤ 0.05. Correlations were estimated using Spearman correlation analysis. The data was analyzed with Statistica software v. 10.0 (StatSoft Inc., Tulsa, OK, USA).

## 3. Results

### 3.1. NAD(P)H Oxidoreductases Activity in Ovarian Cancer Tissues

For each tissue sample, five indices were calculated: the intensity of basic luminescence *I*_0_, the intensity of stimulated luminescence *I*_NADH_ and *I*_NADPH_, and the activation coefficients *K*_NADH_ = (*I*_NADH_ − *I*_0_)/*I*_0_ and *K*_NADPH_ = (*I*_NADPH_ − *I*_0_)/*I*_0_ (Table 1).

**Figure 2:**
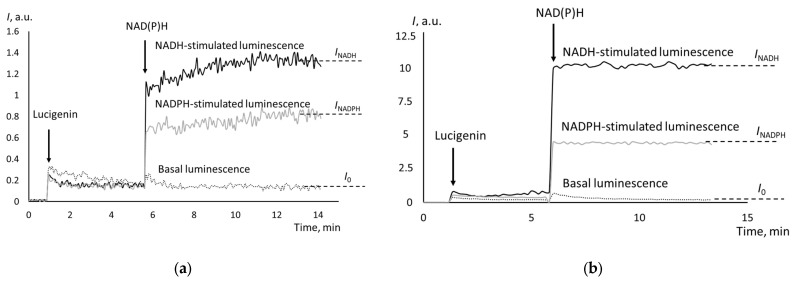
Chemiluminograms of tissue samples of (**a**) cystadenoma and (**b**) moderately differentiated adenocarcinoma; the moments of adding lucigenin and the stimuli are indicated with arrows.

The examples of chemiluminograms for cystadenoma and moderately differentiated adenocarcinoma are presented in Figure 2.

The basal (non-stimulated) chemiluminescence was significantly higher for poorly differentiated adenocarcinomas. For all indices characterizing the activity of CYB5R and CYPOR, no differences were found between cystadenomas and well-differentiated adenocarcinomas, as well as between moderately and poorly differentiated adenocarcinomas. Moderately and poorly differentiated adenocarcinomas are characterized by the significantly higher activity of CYBR5 and CYPOR compared to the control group and well-differentiated adenocarcinomas (see Table 1). The CYB5R activity was higher than the CYPOR activity in almost all cases. There was a direct moderate correlation between the CYB5R and CYPOR activity (*r*_S_ = 0.57).

Note that our assay involves adding an excessive amount of NADH or NADPH; thus, we determine the maximum possible enzyme activity. Reduced levels of NADH or NADPH in cells will likely lead to reduced enzyme activity under real conditions, and elucidating this phenomenon requires separate, complex studies.

### 3.2. CYB5R and CYPOR Activity and Chemoresistance

Since CYPOR is involved in the metabolism of anticancer drugs, the patients with moderate and poor adenocarcinoma were selected, who received paclitaxel and carboplatin A before surgery. The first subgroup included patients without chemoresistance (*n* = 8), and the second subgroup included patients with chemoresistance (*n* = 6). The activity of both CYB5R and CYPOR was significantly lower for the chemoresistant subgroup (Table 2). As for resistant and non-resistant tumors, there was a significant difference in the activity of NAD(P)H-oxidoreductases; this indicator can potentially be used to prognose chemoresistance.

### 3.3. Antioxidant Capacity of Peritoneal Fluid

Reference values for peritoneal fluid could not be determined for ethical reasons, so we compared the subgroups of cancer and benign tumors (cystadenoma). This approach has limitations, since, in benign tumors, the oxidative metabolism of peritoneal fluid can change. However, for benign tumors, the antioxidant capacity of peritoneal fluid remained within the reference values for blood 195–405 a.u. For ovarian cancer, this index was significantly higher. Moderately and poorly differentiated adenocarcinomas were characterized by a significantly higher water-soluble antioxidant capacity than the capacity for well-differentiated adenocarcinomas (Table 3).

**Figure 3:**
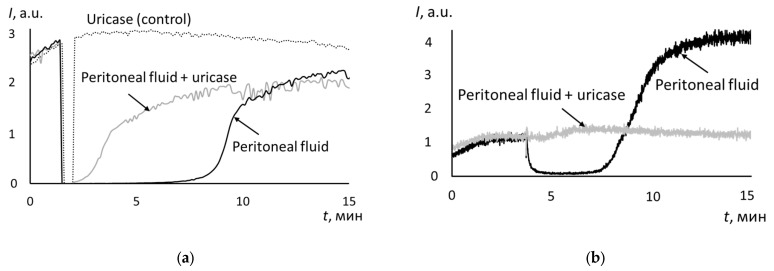
Effect of uricase on the antioxidant capacity of peritoneal fluid of the patient with poorly differentiated adenocarcinoma (**a**) and in the case of cystadenoma (**b**). The dotted line is for uricase without peritoneal fluid (the control sample), the black line is for the peritoneal fluid, and the gray line is for the peritoneal fluid with uricase.

In blood plasma, uric acid provides the main part of the antioxidant capacity *S* [38]. The increase in antioxidant capacity in the peritoneal fluid in ovarian cancer may be due to uric acid or antioxidant metabolites of the tumor tissue. The addition of uricase to the peritoneal fluid of patients with moderately and poorly differentiated adenocarcinomas led to the incomplete suppression of antioxidant activity. As an example, a chemiluminogram is shown for poorly differentiated adenocarcinomas (Figure 3a). For moderately differentiated adenocarcinomas, the chemiluminograms had a similar shape. For peritoneal fluid in cystadenomas and well-differentiated adenocarcinomas, uricase completely neutralized uric acid (Figure 3b). For highly differentiated adenoma, chemiluminograms had a similar shape as in Figure 3b.

The ‘extra’ (non-urate) antioxidant capacity of peritoneal fluid in moderate and poorly differentiated ovarian cancer may be provided by metabolites of the tumor. From the other hand, the peritoneal fluid of patients with ovarian cancer may contain substances that inactivate uricase. Studying this phenomenon needs special experiments.

### 3.4. CYB5R and CYPOR Activity in Cells Isolated from Peritoneal Fluid

The NADH- and NADPH-stimulated chemiluminescence of supernatants after centrifuging the peritoneal fluid samples did not differ from the background signals. Thus, the lucigenin-dependent chemiluminescence in peritoneal fluid is produced only by cells.

**Figure 4:**
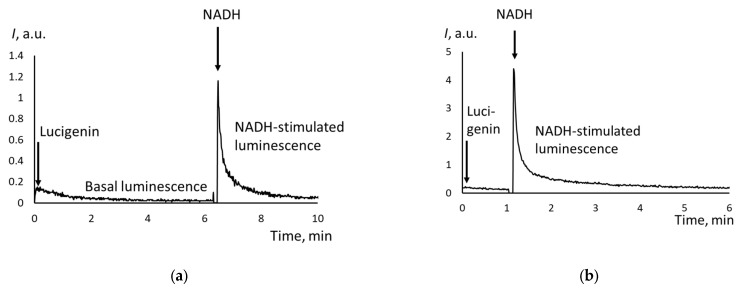
NADH-stimulated chemiluminescence cells from peritoneal fluids from the patients with (**a**) cystadenoma and (**b**) moderately differentiated adenocarcinoma; the moments of adding lucigenin and NADH are indicated with arrows.

The chemiluminograms for cells isolated from peritoneal fluid differed from the chemiluminograms of tissues. For cells, we recorded peak-shaped signals (Figure 4).

Same for tissue samples, the intensity of the NADH-dependent chemiluminescence for cells was slightly higher than that of the NADPH-dependent chemiluminescence. There was a direct moderate correlation between CYB5R and CYPOR activity (*r*_S_ = 0.49).

We did not have a cell sorter device to analyze only ovarian cancer cells, so we analyzed all cells considering these experiments as preliminary and taking into account the fact that ascitic fluid contains mainly cancer cells. For the same reason, we do not present the calculated indices, since they characterize all cells from the peritoneal fluid, and not just ovarian cancer cells. However, the Spearman correlation coefficients between parameters for peritoneal fluid and tissue may give important information (Table 4).

From these data, it follows that there is a direct moderate correlation between the activity of CYB5R and CYPOR in cells isolated from peritoneal fluid and tissues. For benign and well-differentiated tumors, we do not see a correlation between the reductase activity in cells and the antioxidant capacity of peritoneal fluid. However, for moderately and poorly differentiated adenocarcinomas, we see a direct moderate correlation. The higher the activity of reductases, the higher the antioxidant capacity of the peritoneal fluid. It can be assumed that these parameters are related to each other.

## 4. Discussion

The main results of the study are as follows: (1) the activity of NAD(P)H oxidoreductases was lower for benign or well-differentiated tumors compared to moderate or poorly differentiated adenocarcinomas; (2) for chemoresistant adenocarcinomas, CYB5R and CYPOR activity was significantly lower than the activity for non-resistant tumors; (3) for moderately and poorly differentiated adenocarcinomas, the antioxidant capacity of the peritoneal fluid was significantly higher than the capacity for benign and well-differentiated tumors; (4) the NADH- and NADPH-dependent chemiluminescence can also be measured for cells isolated from peritoneal fluid, with a peak-shaped signal; and (5) there is a direct moderate correlation between the antioxidant capacity of the peritoneal fluid and the activity of CYB5R and CYPOR of cells isolated from the peritoneal fluid for moderately and poorly differentiated adenocarcinomas.

NADH-dependent cytochrome b5 reductase is located in the cytoplasmic membrane, and membranes of the endoplasmic reticulum and mitochondria, peroxisomes, nuclei, and sarcoplasmic reticulum. This redox chain is involved in the oxidation of xenobiotics and carcinogens, metabolism of antitumor drugs, elongation of fatty acids, and cholesterol metabolism. CYB5R transfers electrons also to other acceptors, maintaining reduced coenzyme Q10 and ascorbate. Thus, NADH-dependent cytochrome b5 reductase can be considered as a part of the intracellular antioxidant defense [39]. On the other hand, in the oxidation of xenobiotics, ROS are formed, which lead to lipid peroxidation and DNA damage [40]. Thus, the increased activity of CYB5R in the cytoplasmic membrane leads to the excessive production of the superoxide anion radical, which induces irreversible apoptosis in cerebellar neurons [41]. The CYB5R expression in HeLa cells was inhibited by H_2_O_2_ but then increased by an unknown mechanism [42]. Thus, NAD(P)H oxidoreductases play a paradoxical role in the oxidative balance. On the one hand, they are reductants maintaining intracellular antioxidants in a reduced state. On the other hand, they are sources of ROS. This explains the complexity of understanding their role in cell metabolism.

Information on the role of CYB5R in carcinogenesis is very scarce and contradictory. On one hand, CYB5R2 had a tumor-suppressive effect due to the suppression of neoangiogenesis [43]. On the other hand, CYB5R3 played a key role in the extravasation and colonization of cancer cells in mice [16]. The knockdown of this gene led to a significant decrease in tumor mass in the lungs of mice. The authors attributed this effect to influences on TGF-β-dependent signaling pathways, HIF-α-dependent pathways, and apoptosis. Our studies showed that, in papillary thyroid cancer, there are cases with a low and high activity of microsomal reductases, while the cases with high activity were morphologically more unfavorable [34].

The NADPH-dependent cytochrome P450 chain is the main intracellular source of ROS along with the mitochondrial chain. Defects in this chain lead to severe oxidative stress and apoptosis. The overexpression of CYPOR leads to excess ROS production [44]. As a consequence, the functioning of this chain is strictly controlled by the gene expression, protein interactions, and oxidative stress via feedback mechanisms [45]. ROS produced by this chain play an important role in carcinogenesis [46].

In our experiments, the lower activity of NAD(P)H oxidoreductases corresponded to highly differentiated tumors. In moderately and poorly differentiated tumors, reductase activity was the maximum, which probably is a response to intracellular oxidative stress. Interestingly, no differences were found in the cystadenoma and well-differentiated adenocarcinoma groups. In our study, cystadenomas were papillary cysts, which can transform into malignant tumors. This may explain the similar activity of the reductases.

The mechanism of changes in NAD(P)H oxidoreductase activity in cancer is complex and unknown. We studied the gene expression using the RT-PCR method (reverse transcription polymerase chain reaction) and the protein expression using flow cytometry for the same samples of ovarian tissues [47]. In moderate and poor adenocarcinomas, the expression of the CYB5R3 gene, but not CYB5R1 and CYB5R2/4, was decreased. The decrease in CYB5R3 gene expression may be the result of negative feedback in response to the increased activity of the enzyme. The cytochrome P450 reductase activity, on the contrary, was positively correlated with CYPOR gene expression. Thus, the expression of both cytochrome b5 reductase and cytochrome P450 reductase is at least partially regulated at the gene level. The effect of gene expression does not exclude the possibility of other epigenetic causes, the examination of which requires a separate study.

The antioxidant capacity of the peritoneal fluid increased in this series, benign < well-differentiated < moderately and poorly differentiated tumors. An increase in antioxidant capacity can be either a compensatory response to oxidative stress or the result of the production of tumor metabolites with strong antioxidant properties. Tumor metabolites may also inactivate uricase in our experiments. The compensatory character of the increase in antioxidant capacity is partially indicated by the correlations. The higher the activity of reductases (and oxidative stress), the higher the antioxidant capacity of the peritoneal fluid. It can be assumed that these indicators are related to each other. A compensatory increase in antioxidant capacity may occur primarily due to an increase in uric acid levels. The hypothesis that uric acid protects against oxidative damage in cancer has been proposed for a long time [48]. Higher uric acid levels were associated with a lower risk of mortality from any cancer [49]. Higher albumin and uric acid levels were associated with a lower risk of breast cancer development and mortality [50].

There are studies that contradict this hypothesis. An increase in uric acid levels was considered a predictor of death in hopelessly ill patients [51]. High levels of uric acid are associated with an increased risk of overall cancer mortality, especially in women, which also does not support the hypothesis of a protective role of uric acid [52].

Presumably, uric acid may promote inflammation both in a crystalline form, which is recognized by TLR4 receptors, and in a soluble form, which penetrates cells and activates MAP kinases and the NF-κB pathway, possibly through NADPH oxidase [53]. Inflammation associated with uric acid promotes malignant transformation, while increased levels of extracellular uric acid promote the survival of cancer cells and stimulate their proliferation, i.e., the development of highly aggressive cancer [54].

Apoptosis is initiated through reactive oxygen species [55]. Antioxidants play a dual role in carcinogenesis. On the one hand, they suppress tumor growth by preventing oxidative damage to DNA. On the other hand, antioxidants promote cell survival [56]. In our experiments, the increased antioxidant potential of peritoneal fluid may be a variant of metabolic reprogramming and protection from apoptosis.

Another reason for changes in the antioxidant capacity of peritoneal fluid may be a relationship with the activity of the anti-inflammatory pathway NRF2. In our previous study, we found that the correlation of NRF2 expression with the antioxidant capacity of the peritoneal fluid was significant (the correlation coefficient for peritoneal fluid 0.67 vs. 0.36 for blood plasma), which indicates a close metabolic relationship in the abdominal cavity [47].

In conclusion, we will discuss the prospects for using the developed assay to study other types of cancer. We previously performed studies of thyroid tumors [34], and cervical and endometrial cancer [35], and determined the diagnostic significance of CYB5R and CYPOR activity in papillary thyroid cancer, cervical cancer, and endometrial adenocarcinoma. Based on the importance of these enzymes in the metabolism of any tissue and the universality of the assay, these studies will be important for any tumors and cells. In each specific case, appropriate experiments can be carried out using the methodology described in the article.

### Limitations

**Figure 5:**
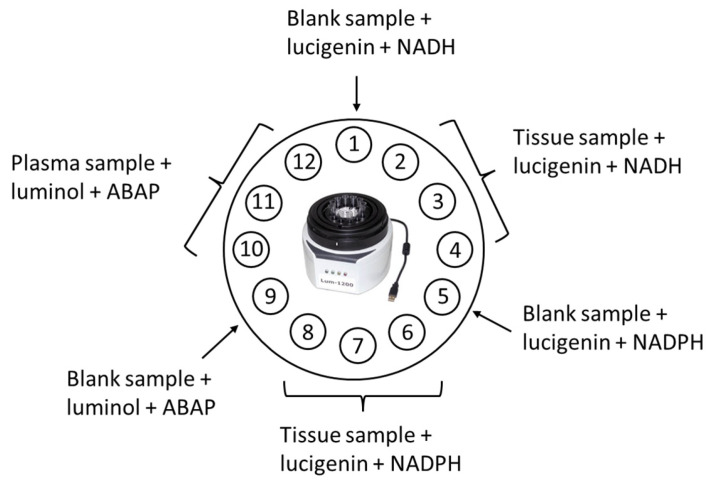
The scheme of simultaneous implementation of protocols for assessment of the activity of CYB5R and CYPOR in tumor tissue samples and water-soluble antioxidant capacity in peritoneal fluid using a Lum-1200 chemiluminometer; buffer solutions were used as control samples; ABAP, 2,2′-azo-bis(2-amidinopropane) dihydrochloride.

Here, we focused on studying only the water-soluble antioxidant potential. Another part of the antioxidant system of biological liquids is represented by lipid-soluble antioxidants, and its study requires an analytical system based on lipid peroxidation. Therefore, to fully characterize the antioxidant potential, information is needed on both the water-soluble and lipid-soluble components. However, our assays for determining the water-soluble antioxidant capacity and NAD(P)H-oxidoreductase activity can be implemented on the same equipment and under the same technical conditions (temperature, sensitivity, and approximately the same recording time) (Figure 5), while the assay for determining lipid antioxidants requires a technically different device with rapid injection. The simultaneous application of two assays will improve the diagnostic and prognostic potential of the method.

Another limitation is that cells isolated from peritoneal fluid samples were not sorted. Therefore, we consider these results as preliminary.

## 5. Conclusions

We applied the developed assay to study the activity of NAD(P)H oxidoreductases based on lucigenin-enhanced and NAD(P)H-stimulated chemiluminescence. The intensity of NADH- and NADPH-dependent chemiluminescence reflects the activity of CYB5R and CYPOR, respectively. This assay can be applied to both tissue samples and cells isolated from peritoneal fluid. For tissues and cells, the activity of CYB5R and CYPOR was significantly higher in moderately and poorly differentiated ovarian adenocarcinomas compared to well-differentiated adenocarcinomas and cystadenomas. The activity of tissue CYB5R and CYPOR was lower for the chemoresistant tumors. In the peritoneal fluid, the antioxidant capacity significantly increased in this series, benign tumors < well-differentiated < moderately and poorly differentiated adenocarcinomas, leading to the antioxidant excess for moderately and poorly differentiated adenocarcinomas. The antioxidant capacity of peritoneal fluid and the activity of CYB5R and CYPOR of cells isolated from peritoneal fluid were characterized by a direct moderate correlation for moderately and poorly differentiated adenocarcinomas. These data indicate the significant role of NAD(P)H oxidoreductases and the antioxidant potential of peritoneal fluid in the biochemistry of ovarian cancer. These indices are useful for diagnostics and prognostics. The developed assay can be used to analyze the CYB5R and CYPOR activity in other tissues and cells.

## Figures and Tables

**Table 1:** The activity of cytochrome b5 reductase and cytochrome P450 reductase in ovarian tumors; * denotes significant differences with the control group (Mann–Whitney test, *p* ≤ 0.05).

Index	Serous Cystadenoma (The Control Group) (*n* = 11), Mean (SD)	Well-Differentiated Adenocarcinoma (*n* = 15), Mean (SD)	Moderately Differentiated Adenocarcinoma (*n* = 13), Mean (SD)	Poorly Differentiated Adenocarcinoma (*n* = 14), Mean (SD)
*I*_0_	0.14 (0.08)	0.20 (0.08)	0.24 (0.18)	0.95 * (0.45)
*I*_NADH_	0.88 (0.55)	0.96 (0.25)	7.28 * (3.55)	8.73 * (3.67)
*K*_NADH_ = (*I*_NADH_ − *I*_0_)/*I*_0_	6.08 (0.96)	5.51 (0.90)	25.83 * (9.41)	21.50 * (10.11)
*I*_NADPH_	0.88 (0.62)	1.71 (1.28)	4.21 * (2.56)	4.18 * (2.23)
*K*_NADPH_ = (*I*_NADPH_ − *I*_0_)/*I*_0_	5.72 (1.25)	8.15 (6.44)	15.30 * (8.02)	12.32 * (7.05)

**Table 2:** The activity of cytochrome b5 reductase and cytochrome P450 reductase in the tissues of non-resistant and chemoresistant moderately and poorly differentiated adenocarcinomas; * denotes significant differences between the subgroups (Mann–Whitney test, *p* ≤ 0.05).

Index	Non-Resistant Adenocarcinoma (*n* = 8), Mean (SD)	Chemoresistant Adenocarcinoma (*n* = 6), Mean (SD)
*I*_NADH_	8.48 (6.12)	4.75 * (2.55)
*I*_NADPH_	7.65 (4.40)	4.42 * (1.94)

**Table 3:** Water-soluble antioxidant capacity of peritoneal fluid for the studied subgroups; * indicates significant differences with the control group (Mann–Whitney test, *p* ≤ 0.05).

Subgroup	Antioxidant Capacity, Mean (SD)
Serous cystadenoma (*n* = 11), the control group	245 (130)
Well-differentiated adenocarcinoma (*n* = 15)	422 * (231)
Moderately differentiated adenocarcinoma (*n* = 13)	658 * (243)
Poorly differentiated adenocarcinoma (*n* = 14)	579 * (260)

**Table 4:** Spearman correlation coefficients between peritoneal fluid and tissue parameters.

Index	*I*_NADH_ (Cells from Peritoneal Fluid)	*I*_NADPH_ (Cells from Peritoneal Fluid)
*I*_NADH_ (tissue)	0.39	—
*I*_NADPH_ (tissue)	—	0.45
Antioxidant capacity of peritoneal fluid for benign and highly differentiated tumors	0.13	0.17
Antioxidant capacity of peritoneal fluid for moderately and poorly differentiated tumors	0.37	0.41

## Data Availability

The raw data supporting the conclusions of this article will be made available by the authors on request.
